# P2Y_6_ receptor‐dependent microglial phagocytosis of synapses mediates synaptic and memory loss in aging

**DOI:** 10.1111/acel.13761

**Published:** 2022-12-24

**Authors:** Jacob M. Dundee, Mar Puigdellívol, Richard Butler, Thomas O. J. Cockram, Guy C. Brown

**Affiliations:** ^1^ Department of Biochemistry University of Cambridge Cambridge UK; ^2^ Institute of Neurosciences University of Barcelona Barcelona Spain; ^3^ The Wellcome Trust Cancer Research UK Gurdon Institute University of Cambridge Cambridge UK

**Keywords:** aging, memory, microglia, P2Y_6_ receptor, phagocytosis

## Abstract

Aging causes loss of brain synapses and memory, and microglial phagocytosis of synapses may contribute to this loss. Stressed neurons can release the nucleotide UTP, which is rapidly converted into UDP, that in turn activates the P2Y_6_ receptor (P2Y_6_R) on the surface of microglia, inducing microglial phagocytosis of neurons. However, whether the activation of P2Y_6_R affects microglial phagocytosis of synapses is unknown. We show here that inactivation of P2Y_6_R decreases microglial phagocytosis of isolated synapses (synaptosomes) and synaptic loss in neuronal–glial co‐cultures. In vivo, wild‐type mice aged from 4 to 17 months exhibited reduced synaptic density in cortical and hippocampal regions, which correlated with increased internalization of synaptic material within microglia. However, this aging‐induced synaptic loss and internalization were absent in P2Y_6_R knockout mice, and these mice also lacked any aging‐induced memory loss. Thus, P2Y_6_R appears to mediate aging‐induced loss of synapses and memory by increasing microglial phagocytosis of synapses. Consequently, blocking P2Y_6_R has the potential to prevent age‐associated memory impairment.

## Graphical abstract

P2Y_6_ receptor (P2Y_6_R) is involved in the increased phagocytosis of synaptic material by microglia, and subsequent memory loss, during aging. P2Y_6_R knockout mice do not exhibit the memory or synaptic loss seen in wild‐type mice.

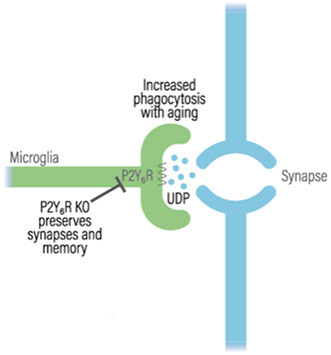

## 1 INTRODUCTION

Brain aging in humans and rodents is accompanied by brain atrophy, loss of synapses, neuroinflammation, and reduced memory and executive functions (Bishop et al., 2010; Cizeron et al., 2020; Morrison & Baxter, 2012; Petralia et al., 2014). Human age‐associated memory impairment is common, affecting about 50% of those older than 60 years, and results in reduced well‐being, function, and economic activity (Koivisto et al., 1995; Larrabee & Crook, 1994). However, the underlying mechanisms causing these changes are unclear, and there are no effective treatments to prevent, delay, or ameliorate aging‐related brain dysfunction.

Microglia are central nervous system macrophages, specialized in the phagocytosis (i.e., engulfment and degradation) of bacteria, synapses, neurons, debris, and aggregated proteins (Gabandé‐Rodríguez et al., 2020; Sierra et al., 2013; Tay et al., 2018; Wolf et al., 2017). Microglia can phagocytose synapses during development, neuropathology, and aging (Butler et al., 2021; Shi et al., 2015; Tay et al., 2018), and microglia can also phagocytose dendrites, axons, and intact neurons (Puigdellívol et al., 2021; Vilalta & Brown, 2018). Microglial phagocytosis of neuronal structures is mediated by eat‐me signals, opsonins, and phagocytic receptors (Butler et al., 2021). Interestingly, it has been shown that genetic knockout of the opsonin C3 reduced aging‐induced loss of hippocampal synapses, neurons, and memory (Shi et al., 2015); and similarly, knockout of the phagocytic receptor TREM2 reduced aging‐induced hippocampal synaptic and neuronal loss in mouse (Linnartz‐Gerlach et al., 2019). Thus, one may hypothesize that the neuroinflammation accompanying aging drives microglial phagocytosis of synapses, resulting in memory impairment and brain atrophy. Microglial biology changes with age, including upregulated expression of phagocytic receptors and opsonins, potentially resulting in excessive phagocytosis of the aging brain (Linnartz‐Gerlach et al., 2019; Shi et al., 2015; Tay et al., 2018).

The P2Y_6_ receptor (P2Y_6_R, expressed from the *P2ry6* gene) is a microglial receptor that mediates microglial phagocytosis of neurons (Koizumi et al., 2007; Neher et al., 2014). P2Y_6_R is expressed by multiple cell types in the body, but within the brain is almost exclusively expressed by microglia (Koizumi et al., 2007; Moore et al., 2001). Damaged or stressed neurons release the nucleotide UTP, which is rapidly degraded into UDP by extracellular nucleotide‐degrading enzymes, and localized UDP then activates the P2Y_6_R on microglia to engulf such neurons (Koizumi et al., 2007). We have shown that activating P2Y_6_R causes microglia to engulf live neurons, and P2Y_6_R deficiency prevents lipopolysaccharide (LPS)‐induced microglial phagocytosis of neurons both in vitro and in vivo (Milde et al., 2021; Neher et al., 2014). Moreover, P2Y_6_R knockout mice were also resistant to memory loss induced by beta‐amyloid and extracellular TAU (Puigdellívol et al., 2021). These previous studies lead us to ask whether (i) P2Y_6_R mediates microglial phagocytosis of synapses, and (ii) the synaptic and memory loss induced by natural aging of mice was also mediated by P2Y_6_R.

We found that aging wild‐type mice to 17 months of age resulted in synapse and memory loss, whereas P2Y_6_R knockout mice had preserved memory. Microglia from 17‐month‐old wild‐type mice had an age‐associated increase in the internalization of synaptic material, but no such increase was observed in microglia from 17‐month‐old knockout mice. Moreover, we show here that inactivation of P2Y_6_R decreases microglial phagocytosis of isolated synapses (synaptosomes) and synaptic loss in neuronal–glial co‐cultures. These findings are significant as they support the hypothesis that microglial phagocytosis of synapses contributes to aging‐induced memory loss, and, more specifically, that inhibition of the P2Y_6_R may prevent this memory loss.

## 2 RESULTS

### 2.1 Genetic and pharmacologic inactivation of P2Y_6_R prevents microglial phagocytosis of isolated synapses

**FIGURE 1:**
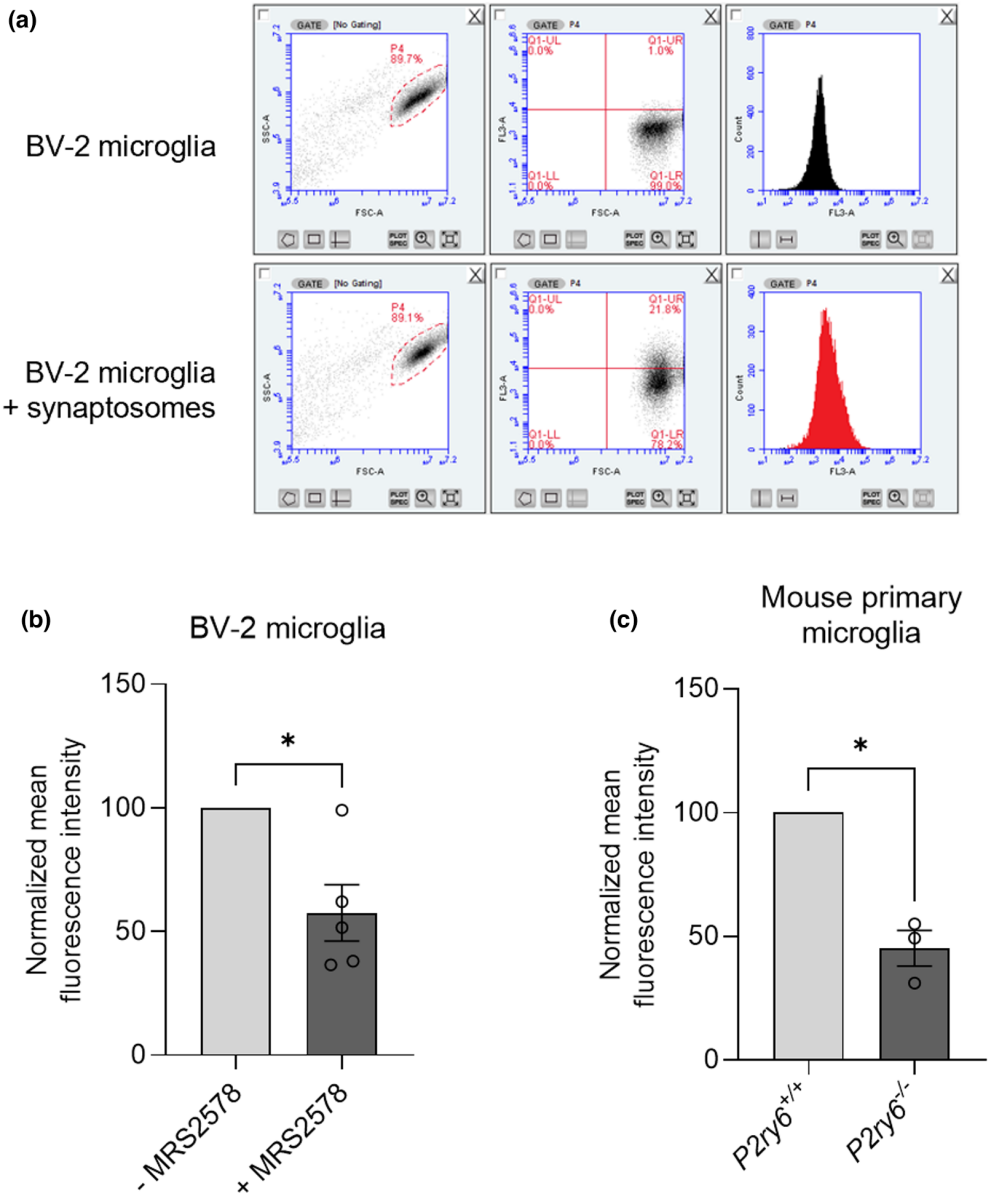
Microglial phagocytosis of synaptosomes is P2Y_6_R dependent. (a) Microglial uptake of synaptosomes was measured by flow cytometry. Microglial events were gated (left) by forward scatter (FSC‐A) and side scatter (SSC‐A), and gated events were analyzed for red fluorescence in FL3 in terms of fluorescence gate shift (middle) or mean fluorescence shift (right). (b) pHrodo‐labeled synaptosome uptake by BV2 microglia (*n* = 5) when treated with 500 nM MRS2578, a P2Y_6_R inhibitor. (c) pHrodo‐labeled synaptosome uptake by *P2ry6* ^*+/+*^ and *P2ry6* ^*−/−*^ primary mouse microglia (*n* = 4). Each point represents one individual experiment with three technical replicates. Statistical comparisons were made via a one‐sample *t* test. Error bars represent ±SEM, **p < 0.01, *p < 0.05

In order to test whether P2Y_6_R regulates microglial phagocytosis of synapses, we measured microglial phagocytosis of isolated synapses (synaptosomes) by flow cytometry. Thus, pHrodo‐labeled synaptosomes were incubated with microglia and the phagocytic uptake of synapses into microglia was measured by flow cytometry (Figure 1a). As a first approach, we used the v‐raf/v‐myc immortalized murine microglial BV2 cell line. BV2 microglia were treated with 500 nM MRS2578, a specific inhibitor of P2Y_6_R (Mamedova et al., 2004), and incubated with pHrodo‐labeled synaptosomes. We found that synaptosome uptake was significantly reduced when P2Y_6_R was pharmacologically inactivated (Figure 1b). We also observed that P2Y_6_R inhibition did not affect the degradation of synaptosomes in BV2 microglia (Figure S1). To test the role of P2Y_6_R genetically in a more physiological model, we used the same experimental approach as for BV2 microglial cells, but using microglial cultures isolated from wild‐type and P2Y_6_R knockout mice. We found decreased phagocytosis of synaptosomes in P2Y_6_R knockout microglia compared with wild‐type (Figure 1c). Altogether, these results indicate that microglial phagocytosis of synaptosomes is P2Y_6_R‐dependent.

### 2.2 P2Y_6_R knockout prevents LPS‐induced synaptic loss in glial‐neuronal cultures

**FIGURE 2:**
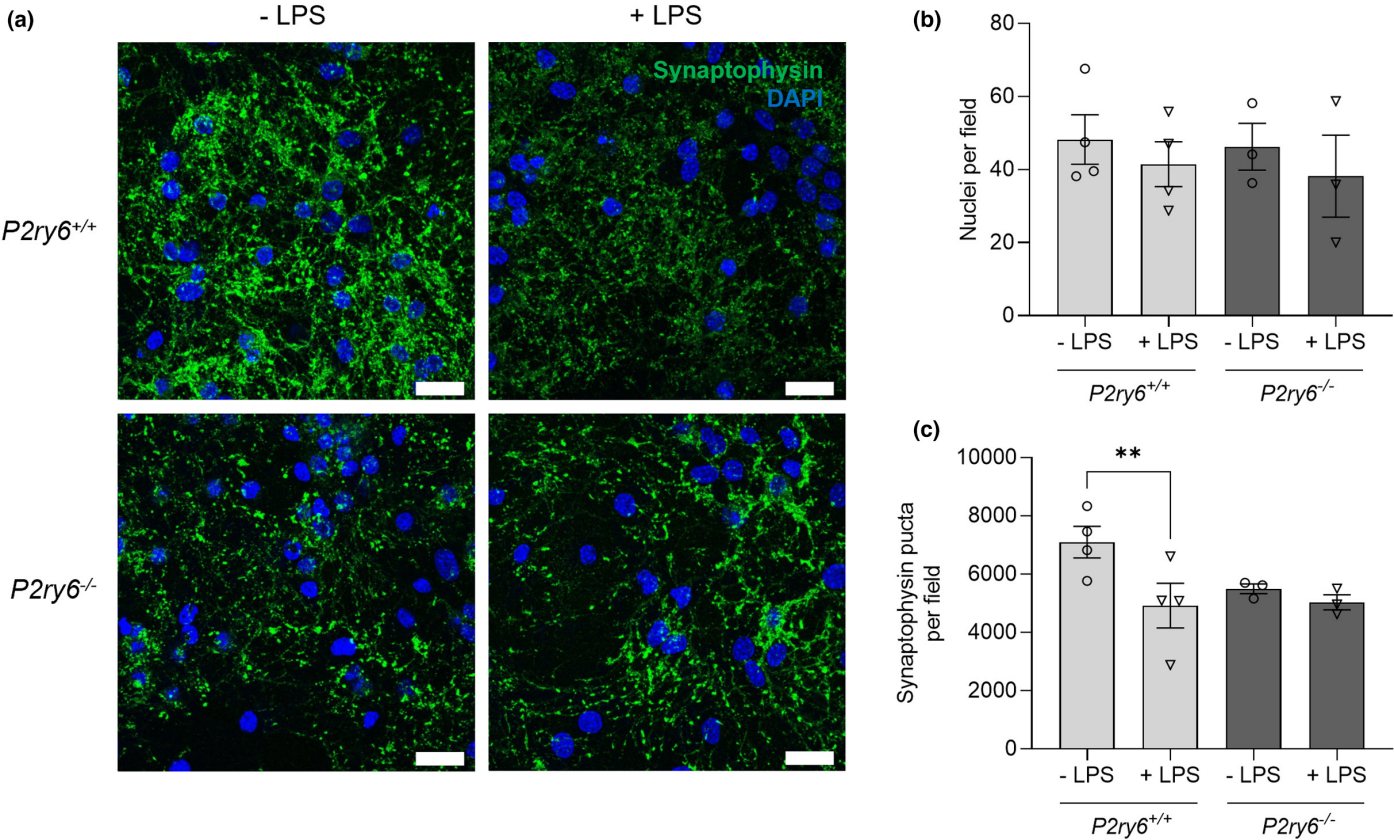
P2Y_6_R deficiency prevents LPS‐induced synaptic loss in glial–neuronal co‐cultures. (a) Representative microscopy images from *P2ry6* ^*+/+*^ and *P2ry6* ^*−/−*^cerebellar glial–neuronal cultures, in the presence of vehicle or 10 ng/ml LPS for 72 h, stained for synaptophysin (green, synaptic marker) and with DAPI (blue, nuclear marker). Scale bar = 25 μm. (b) Nuclei per field measured by counting (*n* = 3–4). (c) Synaptophysin puncta per field were measured using the Fiji plugin Trackmate (*n* = 3–4). Each point represents one individual experiment with three technical replicates. Statistical comparisons were made via a repeated measure two‐way ANOVA with Bonferroni's post hoc comparisons. Error bars represent ±SEM, **p < 0.01.

In order to investigate the role of P2Y_6_R in microglial‐induced synaptic loss, we used a culture model of neuroinflammation induced by LPS, which induces microglial phagocytosis of neurons or synapses (Neher et al., 2014; Sheppard et al., 2019). Age‐associated synaptic loss is closely tied with increased neuroinflammation during aging (Barrientos et al., 2015; Bettio et al., 2017), potentially leading to an increase in microglial phagocytosis (Butler et al., 2021). We treated glial–neuronal cultures from mouse cerebellum with either 10 ng/ml LPS or vehicle for 72 h and then analyzed the density of synaptophysin puncta (as a measure of synapses) by confocal microscopy and image analysis (Figure 2a). To rule out that changes in synaptophysin clusters were due to neuronal loss or death, we first analyzed neuronal density by counting the number of nuclei and found that 10 ng/ml LPS treatment did not induce a significant change in neuronal density (Figure 2b). However, the density of synaptophysin puncta after 10 ng/ml LPS treatment was significantly reduced in P2Y_6_R wild‐type glial–neuronal cultures (Figure 2c), indicating a loss of synapses. In P2Y_6_R knockout cultures, the density of synaptophysin puncta was non‐significantly lower in the absence of LPS, and LPS induced no synaptic loss in P2Y_6_R knockout cultures (Figure 2c). Thus, the LPS‐induced synaptic loss in these cultures was prevented by P2Y_6_R knockout.

### 2.3 P2Y_6_R knockout prevents age‐associated synaptic loss

**FIGURE 3:**
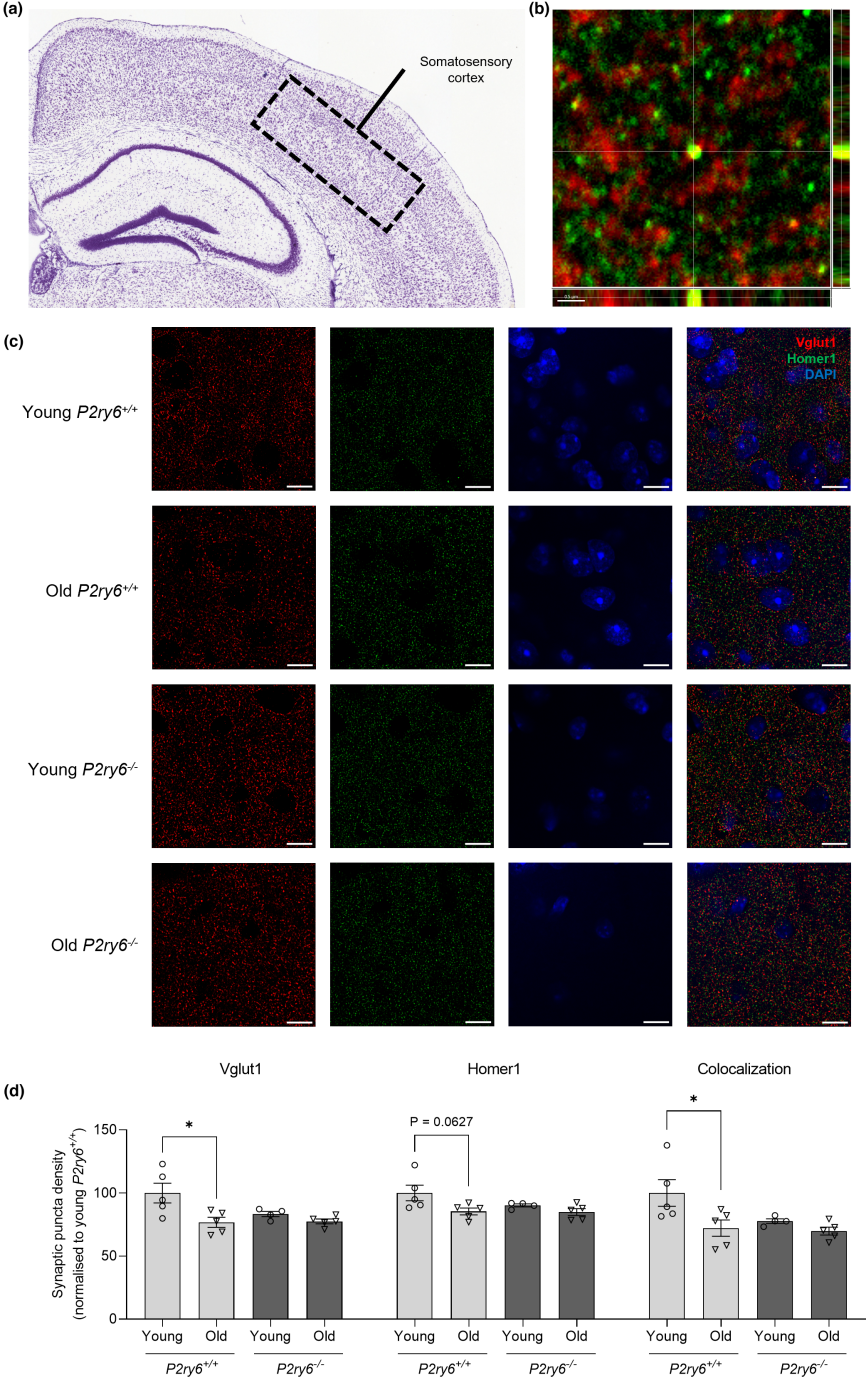
P2Y_6_R deficiency prevents age‐associated synaptic loss in the somatosensory cortex. (a) Nissl stain from the Allen Mouse Brain Atlas and Allen Reference Atlas—Mouse Brain of a coronal section of the mouse brain, with the somatosensory cortex labeled. Available from: mouse.brain‐map.org/static/atlas. (b) Colocalization of Vglut1 and Homer1 puncta. Scale bar = 0.5 μm. (c) Representative confocal microscopy images of young (4 months) and old (17 months) wild‐type and P2Y_6_R knockout mice stained for Vglut1 (red, pre‐synaptic marker), Homer1 (green, post‐synaptic marker), and with DAPI (blue, nuclear stain) in the somatosensory cortex. Scale bar = 5 μm. (d) Vglut1 puncta density, Homer1 puncta density, and synaptic density of the somatosensory cortex (*n* = 4–5, 3 equidistant planes 300 μm apart per mouse). Synaptic density determined as colocalized Vglut1 and Homer1 puncta (<200 nm). Each point represents one animal. Statistical comparisons were made via a two‐way ANOVA with Bonferroni's post hoc comparison test. Error bars represent ±SEM, *p < 0.05

**FIGURE 4:**
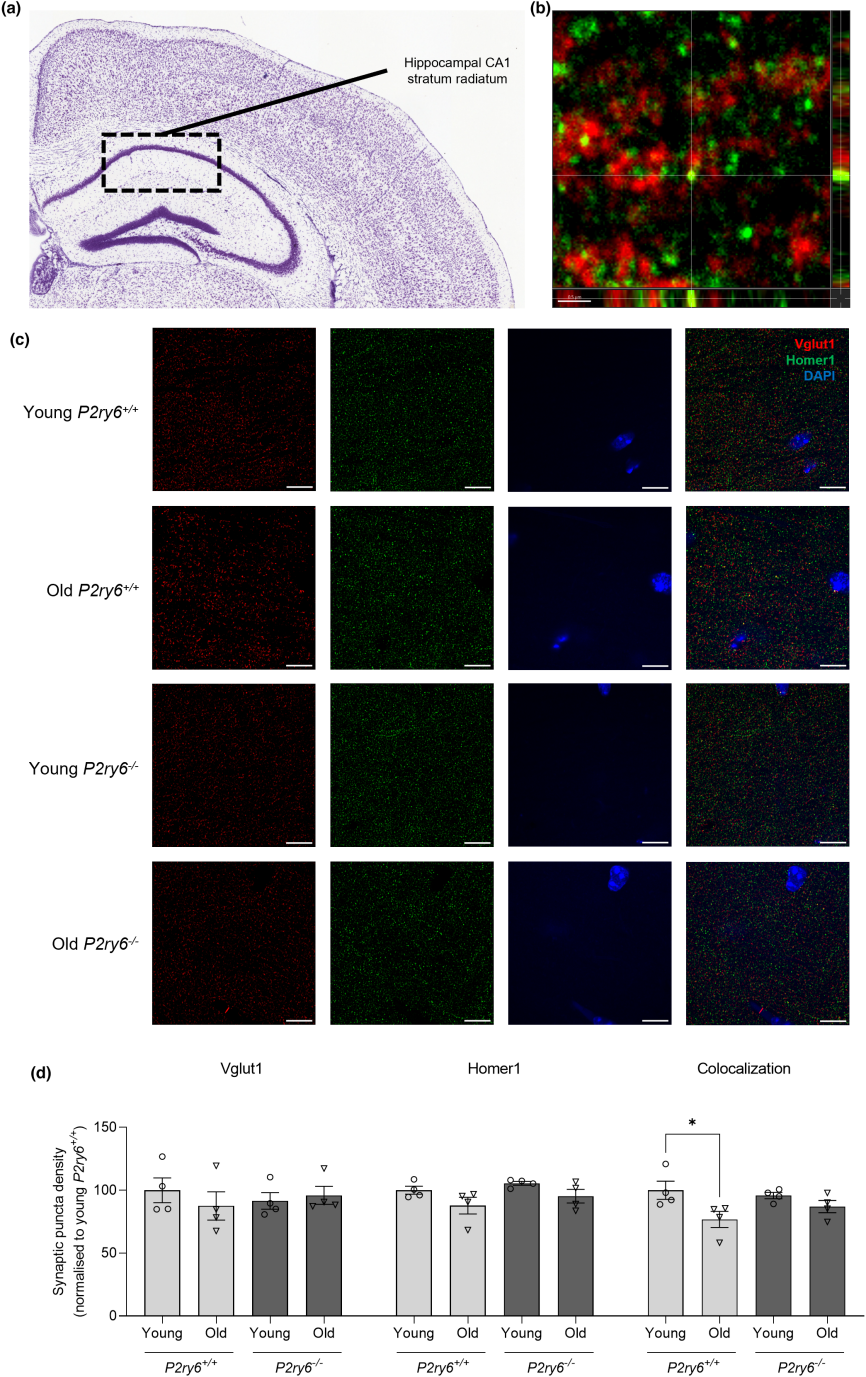
P2Y_6_R deficiency prevents age‐associated synaptic loss in the CA1 hippocampus. (a) Nissl stain from the Allen Mouse Brain Atlas and Allen Reference Atlas—Mouse Brain of a coronal section of the mouse brain, with the hippocampal CA1 stratum radiatum labeled. Available from: mouse.brain‐map.org/static/atlas. (b) Colocalization of Vglut1 and Homer1 puncta. Scale bar = 0.5 μm. (c) Representative confocal microscopy images of young (4 months) and old (17 months) wild‐type and knockout mice stained for Vglut1 (red, pre‐synaptic marker), Homer1 (green, post‐synaptic marker), and with DAPI (blue, nuclear stain) in the somatosensory cortex. Scale bar = 5 μm. (d) Vglut1 puncta density, Homer1 puncta density, and synaptic density of the hippocampal CA1 stratum radiatum (*n* = 4, 3 equidistant planes 300 μm apart per mouse). Synaptic density determined as colocalized Vglut1 and Homer1 puncta (<200 nm). Each point represents one animal. Statistical comparisons were made via a two‐way ANOVA with Bonferroni's post hoc comparison test. Error bars represent ±SEM, *p < 0.05

In order to investigate whether P2Y_6_R can regulate synaptic loss in vivo, we examined aging‐associated synaptic loss in wild‐type and P2Y_6_R knockout mice. For this purpose, synaptic density was analyzed in the somatosensory cortex and the hippocampal CA1 stratum radiatum of young (4 months) and old (17 months) P2Y_6_R wild‐type and knockout mice using confocal microscopy (Figures 3a and 4a). Coronal brain slices were immunostained using antibodies to Vglut1 (pre‐synaptic marker) and Homer1 (post‐synaptic marker), and synaptic density was measured as colocalized (<200 nm) puncta of both synaptic markers in an entire Z‐stack. In the somatosensory cortex, the density of Vglut1 puncta and the colocalization of Vglut1 and Homer1 puncta was significantly decreased in old compared with young wild‐type mice (Figure 3b–d), indicating a significant loss of synapses with age in wild‐type mice. Remarkably, there was no significant age‐dependent decrease in the density of synapses in P2Y_6_R knockout mice, indicating that inactivation of P2Y_6_R attenuates the age‐dependent loss of synapses. There was a trend for reduced synaptic density in young P2Y_6_R knockout mice relative to young wild‐type mice, but this was not significant (Figure 3d, *p* = 0.153). In the CA1 hippocampus, a non‐significant trend to decrease was observed in the density of pre‐ and post‐synaptic clusters with age, but similar to what we found in the somatosensory cortex, colocalization of Vglut1 and Homer1 puncta was significantly decreased with age in wild‐type mice, indicating that synapses in the hippocampal layer are reduced with age (Figure 4b–d). However, as in the somatosensory cortex, there was no decrease in hippocampal synaptic density with age in the P2Y_6_R knockout mice. Altogether, these data indicate that P2Y_6_R is required for the loss of cortical and hippocampal synapses during aging.

### 2.4 P2Y_6_R knockout prevents age‐associated synaptic phagocytosis

**FIGURE 5:**
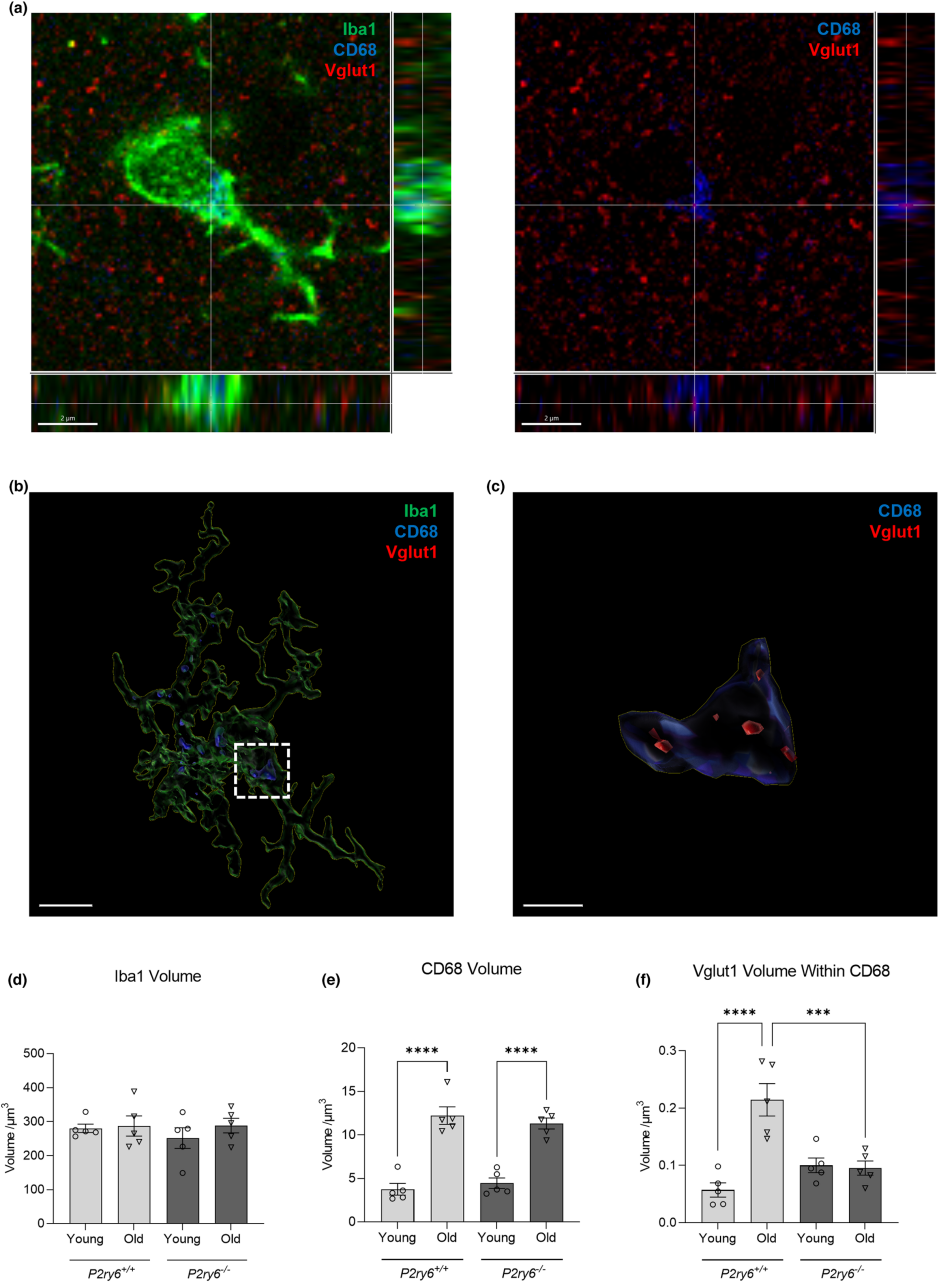
P2Y_6_R deficiency prevents age‐associated synaptic phagocytosis. (a) Representative confocal microscopy image of mice stained for Iba1 (green, microglial marker), CB68 (blue, lysosomal marker), and Vglut1 (red, synaptic marker) in the somatosensory cortex. Scale bar = 2 μm. (b) Representative surface‐rendered microglia (from a). Scale bar = 3 μm. (c) Enlarged inset of Vglut1 colocalization within CD68, denoted by the white dotted line (from b). Scale bar = 0.5 μm. Microglial volume (d), CD68 volume within microglia (e), and Vglut1 colocalization within CD68 (f) across young (4 months) and old (17 months) wild‐type and knockout mice. Each point represents one animal comprised of 13–15 microglia analyzed across three equidistant sections. Statistical comparisons were made via a two‐way ANOVA with Bonferroni's post hoc comparison test. Error bars represent ±SEM, ***p < 0.001, ****p < 0.0001

Age‐associated synaptic loss may be due to increased phagocytosis of synapses, as microglial P2Y_6_R is involved in the formation of the phagocytic cup (Koizumi et al., 2007) triggering the phagocytosis of neuronal material (Milde et al., 2021; Puigdellívol et al., 2021). Thus, we analyzed the internalization of synaptic material within microglial lysosomes in the somatosensory cortex of young (4 months) and old (17 months) wild‐type and knockout mice by confocal microscopy (Figure 5a). Coronal brain slices were immunostained using Iba1 (microglial marker), CD68 (lysosomal marker), and Vglut1 (pre‐synaptic marker), and internalized Vglut1 volume within lysosomal microglia was analyzed by generating Z‐projection surface renderings using Imaris software (Figure 5b,c). There were no significant differences in microglial Iba1 volume between experimental groups (Figure 5d), indicating no effect of age or P2Y_6_R on microglial size. However, microglial CD68 volume significantly increased in old mice compared with young mice (Figure 5e), indicating a higher phagocytic capacity of microglia with age, independent of genotype. Virtually, all of the CD68 staining was within Iba1‐stained microglia, indicating that this marker of phagocytic lysosomes was relatively specific to microglia. Interestingly, the volume of Vglut1 internalized within microglial CD68 increased markedly in old wild‐type mice compared with young wild‐type mice, indicating that aging induces microglial phagocytosis of synapses. However, there was no such increase in old P2Y_6_R knockout mice (Figure 5f), indicating that the aging‐induced microglial phagocytosis of synapses does not occur in P2Y_6_R knockout mice. Taken together, these data strongly implicate P2Y_6_R in the age‐associated loss of synapses by phagocytosis.

### 2.5 P2Y_6_R knockout prevents age‐associated memory loss

**FIGURE 6:**
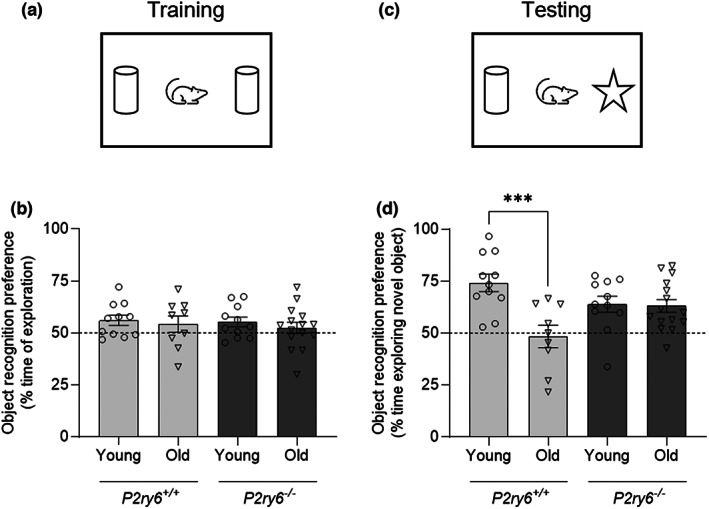
P2Y_6_R deficiency prevents age‐associated memory loss. (a) Schematic representation of the training session in the NORT. (b) Object preference of each animal as percentage of time spent exploring two identical objects (*n* = 9–15). (c) Schematic representation of the testing session in the NORT. (d) Object preference of each animal as percentage of time spent exploring the novel object 24 h after training (*n* = 9–15). Each point represents one animal. Dashed lines indicate a 50% chance level. Statistical comparisons were made via a two‐way ANOVA with Bonferroni's post hoc comparisons test. Error bars represent ±SEM, ***p < 0.001.

Age‐associated synaptic loss correlates with age‐associated memory loss (Cizeron et al., 2020; Linnartz‐Gerlach et al., 2019; Morrison & Baxter, 2012; Shi et al., 2015; Yu et al., 2011), and age‐associated memory loss has been reported to occur in C57BL/6J wild‐type mice as early as 10 months (Magnusson et al., 2003; Wong & Brown, 2007; Yu et al., 2011). We have previously observed that P2Y_6_R knockout mice do not exhibit memory loss in both an acute amyloid model and chronic tau model of neurodegeneration (Puigdellívol et al., 2021). To investigate whether P2Y_6_R has a role in age‐associated memory loss, memory was tested in young (4 months) and old (17 months) wild‐type and P2Y_6_R knockout mice using the novel object recognition test (Figure 6). This is a test evaluating recognition memory (Ennaceur & Delacour, 1988), where mice were habituated in an open field for two consecutive days, and on the third day, mice were allowed to explore two identical objects for 10 min. Twenty‐four hours after training, one of the objects (familiar) was replaced by a new object (novel), and the relative time spent with novel and familiar objects was quantified. During the training session, the mice had no overall preference for either object (Figure 6a). By contrast, 24 h after training, young wild‐type mice spent more time with the novel object, that is, they had long‐term memory of objects. However, old wild‐type mice demonstrated impaired recognition memory as manifested by similar exploration times of the novel and familiar object (Figure 6b). Thus, aged wild‐type mice lose the ability to recognize novel objects. By contrast, P2Y_6_R knockout mice had no significant loss of memory with age (Figure 6b), indicating that genetic inactivation of P2Y_6_R prevents age‐associated memory loss.

## 3 DISCUSSION

We have shown that microglial phagocytosis of isolated synapses is P2Y_6_R dependent, LPS‐induced synaptic loss in culture is P2Y_6_R dependent, age‐associated internalization of synapses into microglia is P2Y_6_R dependent, age‐associated synaptic loss in mice is P2Y_6_R dependent, and age‐associated memory loss in mice is P2Y_6_R dependent. Thus, P2Y_6_R deficiency can prevent age‐associated memory loss (and potentially other cognitive deficits), probably by blocking microglial phagocytosis of synapses, although other mechanisms might contribute.

P2Y_6_R has previously been shown to mediate microglial phagocytosis of neurons as a result of stressed, damaged, or dying neurons releasing the nucleotide UDP into the extracellular space where it can locally activate P2Y_6_R on microglia, inducing microglial phagocytosis of neurons (Koizumi et al., 2007; Neher et al., 2014; Puigdellívol et al., 2021). UDP can be released from stressed or apoptotic cells via pannexin or connexin channels (Elliott et al., 2009; Lazarowski, 2012), and we have shown that, for example, amyloid beta can increase release of UDP (Puigdellívol et al., 2021). Thus, it is possible that stressed synapses may locally release UDP, resulting in their phagocytosis by microglia. Microglia are the main cells expressing P2Y_6_R in the brain (Koizumi et al., 2007; Moore et al., 2001). Extracellular UDP induces microglial phagocytosis via a P2Y_6_R‐dependent inositol triphosphate and calcium rise, which triggers local formation of the phagocytic cup, a late phase in engulfment (Abbracchio et al., 2006; Koizumi et al., 2007).

P2Y_6_R‐dependent microglial phagocytosis might potentially be beneficial by removing debris, plaques, and/or pathogens in the brain, and there are patents suggesting that P2Y_6_R agonists can be beneficial by stimulating microglial phagocytosis of plaques (Haydon et al., 2013; Haydon & Lee, 2014). However, there is no evidence that debris or plaques release UDP, and phagocytosis of debris in vitro is unaffected by P2Y_6_R knockout (Puigdellívol et al., 2021). Therefore, blocking P2Y_6_R should not be detrimental, and this is supported by our findings that P2Y_6_R knockout is beneficial in natural aging (this paper) and models of neurodegeneration (Milde et al., 2021; Puigdellívol et al., 2021).

Our culture experiments included serum, which has been shown to stimulate microglial phagocytosis, in part by supplying opsonins (Bohlen et al., 2017). Thus, it may be informative to test the role of P2Y_6_R in phagocytosis in other culture conditions, particularly those relevant to aging. Note that the aging‐induced synapse loss that we found here was quantified using confocal microscopy on 25 μm sections, which has limitations due to the thickness of these sections (Avila & Henstridge, 2022), and ideally would be checked using array tomography of ultrathin sections. Our in vivo data showed that aging induced a large increase in synaptic markers within CD68+ lysosomes, and this was prevented by P2Y_6_R knockout (Figure 5f). This could have been due to higher lysosomal degradation in the P2Y_6_R knockout. However, the amount of CD68+ lysosomes was the same in the P2Y_6_R knockout and wild‐type mice (Figure 5e), and we found that P2Y_6_R inhibition with MRS2578 had no effect on the kinetics of lysosomal degradation (Figure S1). This suggests that the in vivo data are not due to increased lysosomal degradation when P2Y_6_R is blocked, but rather reduced microglial phagocytosis of synapses.

Microglial phagocytosis of synapses has previously been shown to be mediated by: (i) complement components C1q and C3 binding to unknown factors on synapses to induce complement receptor 3 dependent microglial phagocytosis of the opsonized synapses (Schafer et al., 2012), (ii) phosphatidylserine exposure on synapses, engaging unknown opsonins and phagocytic receptors on microglia (Scott‐hewitt et al., 2020), and (iii) TREM2, as a phagocytic receptor on microglia, potentially binding phosphatidylserine or APOE on synapses (Atagi et al., 2015; Filipello et al., 2018; Scott‐hewitt et al., 2020). Microglial phagocytosis of synapses is also regulated by the don't‐eat‐me signals CD47 and sialic acid on synapses (Lehrman et al., 2018; Linnartz‐Gerlach et al., 2016) and the find‐me signal fractalkine (CX3CR1) released by synapses (Paolicelli et al., 2011). Whether UDP release, stimulating microglial P2Y_6_R, acts as a signal for synapse phagocytosis that is independent of (or dependent on) all these other signals is unclear. Most phagocytic events require multiple signals, and as UDP acts at a relatively late stage of engulfment (formation of the phagocytic cup), it may trigger the final, irreversible stage of engulfment that is initiated by other signals (Cockram et al., 2021).

We found that low‐dose LPS‐induced synaptic loss in glial‐neuronal cultures, that was prevented by P2Y_6_R knockout. It has previously been shown that low‐dose LPS induces synaptic loss in hippocampal slice cultures that is dependent on microglia (Sheppard et al., 2019), and LPS can induce synaptic loss in vivo in mice (Xin et al., 2019). We have previously shown that P2Y_6_R knockout or inhibition can prevent LPS‐induced neuronal loss in mice (Milde et al., 2021; Neher et al., 2014). Thus, our novel finding here that P2Y_6_R knockout prevents LPS‐induced synaptic loss suggests the possibility that pharmacological inhibition of P2Y_6_R might prevent the synaptic loss in diseases characterized by neuroinflammation, such as Alzheimer's disease. We have previously shown that P2Y_6_R knockout prevents the memory loss induced by amyloid beta or P301S TAU, as models of neurodegeneration (Puigdellívol et al., 2021); thus, it is possible that this memory loss was mediated by P2Y_6_R‐dependent microglial phagocytosis of synapses, although this was not tested.

Previous studies have highlighted that age‐associated memory loss follows age‐associated synaptic loss (Morrison & Baxter, 2012) and that microglia remove these synapses through phagocytosis (Shi et al., 2015; Weinhard et al., 2018). Our findings here that P2Y_6_R regulates microglial phagocytosis of synapses, and P2Y_6_R knockout prevents age‐associated synaptic and memory loss, supports the hypothesis that age‐associated synaptic and memory loss is due to microglial phagocytosis of the synapses. In addition, our findings indicate that blocking P2Y_6_R can prevent age‐associated memory loss, so it may be worth testing whether a P2Y_6_R inhibitor can prevent or reverse age‐associated memory loss.

## 4 EXPERIMENTAL PROCEDURES

### 4.1 Animals

All animal work was carried out in accordance with the Animals (Scientific Procedures) Act 1986 Amendment Regulations 2012 following ethical review by the University of Cambridge Animal Welfare and Ethical Review Body (AWERB). P2Y_6_R knockout mice were kindly provided by Bernard Robaye (ULB Brussels) and maintained on a C57BL/6 background (Charles River Laboratories). P2Y_6_R knockout mice and wild‐type littermates were used to establish homozygous P2Y_6_R wild‐type and knockout sub‐lines.

### 4.2 Immortalized cultures

The v‐raf/v‐myc immortalized murine microglial BV2 cell line (ECACC Cat# 0356, RRID: CVCL_0182) was maintained as described by Blasi et al. (1990). Briefly, cells were cultured in DMEM supplemented with 10% FBS (Gibco). Cells were detached using 0.05% Trypsin–EDTA (Gibco) and centrifuged at 150 RCF for 5 min. Cells were resuspended in DMEM supplemented with 0.5% FBS (Gibco) and seeded in a 24‐well plate at 1 × 10^5^ cells per well and incubated overnight before the phagocytosis assay. All tissue culture medium was supplemented with 100 U/ml penicillin and 100 μg/ml streptomycin (Sigma‐Aldrich). All cells were kept in a humidified incubator at 37°C and 5% CO_2_.

### 4.3 Primary cultures

Primary mixed neuronal/glial cultures were prepared from cerebella of postnatal day 3–5 P2Y_6_R wild‐type and knockout mouse pups as described previously by Puigdellívol et al. (2021). After 7–9 days, the culture composition of these cultures was 85 ± 5% neurons, 7 ± 3% astrocytes, and 5 ± 3% microglia. Primary mouse microglial cells were prepared as described previously by Carrillo‐Jimenez et al. (2018). Briefly, mixed glial cultures were obtained from the cortex of mouse/rat pups (postnatal days 4–7). Isolated primary microglial cultures were obtained by gently vortexing the mixed glial culture for 1 min to detach microglia and centrifuging the supernatant at 150 RCF for 7 min with no brake. The microglia were resuspended in medium consisting of one‐part conditioned media and two parts fresh DMEM supplemented with 10% performance plus FBS (Gibco). The cells were seeded on poly‐l‐lysine‐coated 24‐well plate at 1 × 10^5^ cells per well and incubated overnight before the phagocytosis assay. All tissue culture medium was supplemented with 100 U/ml penicillin and 100 μg/ml streptomycin (Sigma‐Aldrich). All cells were kept in a humidified incubator at 37°C and 5% CO_2_. Cerebellar granular cell (CGC) cultures were treated with either 10 ng/ml LPS or phosphate‐buffered saline (PBS; vehicle).

### 4.4 Synaptosome preparation

Synaptosomes were isolated from rat cortex via the Percoll gradient procedure described by Dunkley et al. (2008). Male rats weighing between 125 and 150 g were culled via cervical dislocation, followed by decapitation to confirm death. Brains were quickly extracted and retained in homogenizing buffer (0.32 M sucrose, 1 mM EDTA, 5 mM Tris, 250 μM DTT, pH 7.4) on ice while cortical slices were generated. Slices were homogenized via 10 firm up‐down motions with a mechanical homogenizer, or until solution had cleared of visible remnants. Homogenate was stored on ice before spinning down via centrifugation (1000 RCF, 10 min, 4°C). The supernatant (S1) fraction was saved and loaded onto prepared Percoll gradients at 2 ml per tube; the pellet was discarded. After loading S1, tubes were spun down using a JA 25.50 rotor (Beckman) at 48,254.4 RCF (4°C, slow start/stop). Spins were timed such that tubes were subjected to precisely 12 min at maximum speed, to ensure proper separation of fractions down the gradient. The synaptosome fraction was then extracted using a glass pipette, with care taken to avoid extracting undesired fractions, and diluted in ~40 ml of ice‐cold homogenizing buffer. To eliminate any contaminating silica aggregates from the Percoll, this synaptosome‐containing solution was spun again using a JA 25.50 rotor (Beckman) at 28,982.8 RCF (4°C, slow‐stop). Supernatant was aspirated, with care taken not to dislodge the synaptosome pellet. The pellet was then resuspended in ice‐cold homogenizing buffer to 1.5 ml and spun using a benchtop centrifuge (Eppendorf) for 10 min (20,000 RCF, 4°C). The synaptosome pellet was finally resuspended to 1 ml with homogenizing buffer containing 5% DMSO, aliquoted, and cryogenically frozen until use. A few microliters were retained and used to measure synaptosome concentration, which was approximated by quantifying protein density at 280 nm using a nanodrop (Thermo Fisher).

### 4.5 Phagocytosis of synaptosomes by flow cytometry

Synaptosomes were thawed in warmed, CO_2_‐infused HBK buffer (HEPES‐buffered Krebs‐like) from cryogenically frozen stocks. Synaptosomes were spun down (20,000 RCF, 5 min), resuspended in warmed HBK buffer, and stained with pHrodo Red succinimidyl‐ester (10 μM) for 15 min in the dark (37°C). pHrodo‐conjugated synaptosomes were then washed with three spin‐resuspension cycles before finally resuspending in HBK buffer. pHrodo‐conjugated synaptosomes were added directly to cells were incubated for 60 min (37°C, 5% CO_2_). After 60 min, microglia were washed with PBS and incubated with PBS containing 0.05% trypsin for 10 min at 37°C, before trypsin was quenched with two parts DMEM containing 10% FBS. Suspended samples were then spun down (150 RCF, 5 min), resuspended in PBS, and retained on ice until analysis via an Accuri C6 flow cytometer (BD Systems). 2500–12,000 events were collected.

### 4.6 Degradation of synaptosomes measured by flow cytometry

Synaptosomes were thawed in warmed, CO_2_‐infused HBK buffer (HEPES‐buffered Krebs‐like) from cryogenically frozen stocks. Synaptosomes were spun down (20,000 RCF, 5 min), resuspended in warmed HBK buffer, added directly to cells, and incubated together for 2 h (37°C, 5% CO_2_). After 2 h, microglia were washed with PBS and incubated with DMEM containing 0.5% FBS at 37°C for 0, 2, or 4 h, with and without 1 μM MRS2578, a P2Y_6_R inhibitor. After the incubation periods, microglia were washed with PBS and fixed with 4% paraformaldehyde (PFA) for 10 min. Fixed cells were then stained with mouse anti‐Vglut1 (1:500, Thermo Fisher, MA5‐31373) in PBS for 1 h. Cells were then washed with PBS and then incubated with Alexa‐Fluor 568 goat anti‐mouse (1:200, Thermo Fisher, A5054) in PBS for 30 min. Cells were washed with PBS and incubated with PBS containing 0.05% trypsin for 10 min at 37°C before trypsin was quenched with two parts DMEM containing 10% FBS. Suspended samples were then spun down (150 RCF, 5 min), resuspended in PBS, and retained on ice until analysis via an Attune NxT Flow Cytometer (Thermo Fisher Scientific). Percentage of Vglut1‐positive cells was gated from unstained BV2 cells and then subtracted from the percentage of stained BV2 cells without the addition of synaptosomes. 2000–3000 events were collected.

### 4.7 Immunocytochemistry of CGC cultures

Immunocytochemistry of CGC cultures was performed as described previously by Puigdellívol et al. (2015). Briefly, after 14 DIV, cells were washed with PBS and fixed with PBS containing 4% PFA for 10 min at room temperature. Cells were washed three times with PBS and blocked using PBS containing 0.1 M glycine for 10 min. After three washes with PBS, cells were permeabilized using PBS containing 0.1% saponin for 10 min, rinsed three times with PBS, and blocked using PBS containing 15% BSA for 30 min. Cells were then washed with PBS and incubated overnight at 4°C with PBS containing 5% BSA, and anti‐synaptophysin antibody (1:300, Abcam, 32127). The next day, cells were washed three times with PBS and incubated with PBS containing 5% BSA, and Alexa‐Fluor 488 anti‐rabbit antibody (1:200, Thermo Fisher, A11008) for 2 h at room temperature. Cells were then washed three times with PBS and mounted on glass slides using Vectashield mounting medium with DAPI (Vector Laboratories, H1500). Slides were stored at 4°C until analysis via confocal microscopy.

### 4.8 Neuronal survival

Cell survival of CGC neurons at DIV14 after vehicle or LPS treatment was assessed by nuclear DNA staining with DAPI. Neurons at DIV14 were fixed with 4% PFA for 10 min at room temperature. Fixed cells were then washed three times in PBS and mounted under glass coverslips with Vectashield mounting medium with DAPI (Vector Laboratories, H1500). Neuronal survival is represented as the number of DAPI‐stained nuclei‐positive cells. Four Z‐stacks of 2.4 μm (0.4 μm steps) were taken per duplicate per condition.

### 4.9 Synaptophysin puncta analysis of CGC cultures

Cerebellar granular cell cultures were imaged using a TCS SP8 confocal microscope (Leica). Coverslips were imaged at 63× magnification, with four images taken per coverslip and Z‐stacks of 2 μm with six steps (0.4 μm per step) per image. All images were analyzed using Fiji (Schindelin et al., 2012); all puncta quantification was done using Trackmate v5.0.1622. For each image, a Z‐project was made (projection type: max intensity) and a gaussian blur was applied (sigma radius: 1). On Trackmate, a DoG detector was used (estimated blob diameter: 0.72 μm), and “mean intensity” and “quality” parameters were chosen based on qualitative assessment of puncta assignment from random example images taken from each experimental condition. Once chosen, the same parameters were used for all images within the same experiment, to ensure no bias between conditions. Data were represented as average synaptophysin puncta number per field from all images of the same condition. DAPI‐stained nuclei were counted manually, and these data were represented as average nuclei number per field from all images of the same condition.

### 4.10 Transcardial perfusion and tissue sectioning

Mice were given terminal anesthesia (150 μl Euthatal intraperitoneal (i.p.)) and, once unresponsive to pain, perfused transcardially, through a 25‐gauge needle, with 20 ml PBS pH 7.4 followed by 60 ml 4% PFA, pH 7.4 using a perfusion pump with a flow rate of 4 ml/min. Following perfusion, brains were removed and post‐fixed overnight in the same solution, cryoprotected by immersion in an increased 10%–30% sucrose solution until sectioning. Serial coronal sections (25 μm) through the whole brain were collected using a sliding microtome and placed in PBS with 0.025% sodium azide as free‐floating sections.

### 4.11 Immunohistochemistry of free‐floating brain slices

All steps were carried out at room temperature, with shaking, and rinsing thrice with PBS after each incubation unless stated otherwise. Five to six free‐floating 25 μm sections taken every 12th brain section of 4–5 P2Y_6_R wild‐type and knockout mice at both 4 and 17 months of age were used for immunohistochemistry. Sections were rinsed three times in PBS and incubated with 50 mM ammonium chloride in PBS for 30 min to quench free aldehyde groups from fixation. Sections were then incubated in 0.1% Sudan Black B in 70% ethanol for 20 min to reduce autofluorescence, permeabilized using 1% Triton X‐100 in PBS for 30 min to facilitate antibody penetration, and blocked for 1 h with blocking solution (2% bovine serum albumin, 3% goat serum, and 0.03% Triton X‐100 in PBS). Subsequently, sections were incubated with mouse anti‐Vglut1 (1:200, Thermo Fisher, MA5‐31373), rabbit anti‐Homer1 (1:500, Synaptic Systems, 160003), rabbit anti‐Iba1 (1:200, Wako, 019‐19741), and rat CD68 (1:200, Thermo Fisher, 14‐0681‐82) antibodies in blocking solution for 2 h at 37°C (Xiao et al., 2017). Sections were then rinsed three times with PBS and then incubated with Alexa‐Fluor 568 goat anti‐mouse (1:200, Thermo Fisher, A5054), Alexa‐Fluor goat 488 anti‐rabbit (1:200, Thermo Fisher, A11008), and Alexa‐Fluor 647 goat anti‐rat (1:200, Thermo Fisher, A21247) antibodies for 2 h at 37°C. Sections were then rinsed three times with PBS and mounted on poly‐l‐lysine‐treated glass slides and dried at 37°C. Sections were then mounted using Vectashield mounting medium with DAPI (Vector Laboratories, H1500) and imaged using confocal microscopy.

### 4.12 Synaptic density analysis of free‐floating brain sections

Imaging was carried out on a Nikon C2si confocal microscope with a 63×, 1.35 NA oil immersion objective using 405, 488, and 561 nm laser lines. A 2‐μm *Z*‐stack (0.125 μm step intervals) was collected being 2–5 μm from the surface of the section at each region of interest. Six to nine images were taken across three sections 300 μm apart per mouse. Background subtraction (six pixels rolling ball) and intensity normalization (2%) across the sections were carried out using Fiji (Schindelin et al., 2012). We developed a custom script for Fiji to map Vglut1 and Homer1 puncta positions in 3D and analyze their distributions. In order to detect puncta 0.2 μm in diameter (Moreno Manrique et al., 2021), the script applies a Laplacian of Gaussian filter with the standard deviation set from this estimated diameter and detects local maxima as puncta candidates. Candidate points are then clustered into final puncta by merging points within one punctum width of each other, excluding points on image edges and below the global Otsu intensity threshold (Otsu et al., 1979). The number of puncta “colocalized” between the two channels is counted as the number of C2 (Homer1) coordinates having at least one C3 (Vglut1) coordinate within one punctum width. Local density is calculated for each punctum using a Gaussian kernel density estimate (Davis et al., 1956), and overall density in each channel is calculated as puncta per μm^3^. The results were normalized to the mean of 4‐month‐old wild‐type mice.

### 4.13 Synaptic internalization analysis of free‐floating brain sections

Imaging was carried out on a Nikon C2si confocal microscope with a 63×, 1.35 NA oil immersion objective using 488, 561, and 640 nm laser lines. Microglia were imaged and analyzed following Schafer et al. (2014). Briefly, Z‐stacks (0.5 μm step intervals) were collected being 2 μm from the surface of the section at each region of interest. Thirteen to 15 microglia were analyzed across three sections 300 μm apart per mouse. Background subtraction (six pixels rolling ball) and intensity normalization (2%) across the sections was carried out using Fiji (Schindelin et al., 2012). Microglial (Iba1), lysosomal (CD68), and synaptic (Vglut1) surface rendering was carried out using Imaris 9.1.2. The results for the surface‐rendered objects were represented as volume (μm^3^).

### 4.14 Novel object recognition test and novel location recognition test

Novel object recognition testing (NORT) was performed in a 30 × 44 cm arena with opaque sides, with a 24‐h retention time to test long‐term memory (Puigdellívol et al., 2021). Briefly, 4‐ and 17‐month‐old mice were first habituated to the arena in the absence of objects on two consecutive days (15 min/day), when spontaneous locomotor activity (total distance traveled) and anxiety/motivation (distance traveled in periphery versus center of the open field) were measured. On the third day, two similar objects were presented for 10 min (A and A' objects). Twenty‐four hours later, the same animals were retested for 5 min in the arena with a familiar (A) and a new (B) object. The object preference was measured as the time exploring each object × 100/time exploring both objects. Animals were tracked and recorded with SMART Junior software (Panlab). Objects and arena were cleaned thoroughly with 70% ethanol and dried after each trial to eliminate odor cues. Experimenter was blinded to the genotype of the individual animals.

### 4.15 Statistical analysis

For in vitro studies, bars represent mean ± SEM, and each data point represents one independent experiment performed in triplicate (Figures 1b,c, 2b,c and S1). For in vivo studies, bars represent mean ± SEM, and each data point represents one animal (Figures 3d, 4d, 5d–f and 6a,b). Statistical differences in Figure 1 were calculated using a one‐sample *t* test. Statistical significance for Figure 2 was analyzed using a repeated measure two‐way ANOVA with Bonferroni's post hoc comparisons. Statistical significance for Figures 3, 4, 5, 6, and S1 were analyzed using an ordinary two‐way ANOVA with Bonferroni's post hoc comparisons. All experiments were analyzed using GraphPad Prism 9 (GraphPad software). Graphical data were shown as individual data points, including mean values with error bars indicating SEM. *p*‐values of *p < 0.05, **p < 0.01, ***p < 0.001 indicated significant differences between groups. For each experiment and graph, statistical details including the statistical test used, the exact value of *n*, what *n* represents (number of animals per genotype, etc.) as well as dispersion and precision measures (mean, SEM, etc.) can be found in each figure legend.

## AUTHOR CONTRIBUTIONS

MP managed the animal colonies and performed the in vivo experiments. JMD analyzed the brain sections. RB helped analyze the brain sections. TOJC and JMD performed and analyzed the experiments in culture. MP and GCB conceived and managed the research. JMD, MP, and GCB wrote the manuscript. All authors reviewed and approved the manuscript.

## CONFLICT OF INTEREST

The authors declare no competing interests.

## Supporting information


Figure S1

Appendix S1

## Data Availability

All code used to analyze synaptic puncta can be found at https://github.com/gurdon‐institute/Synaptic‐Density‐Analysis. Experimental data reported in this paper will be shared by the corresponding authors upon request.
