# Supplementary figures and images for: P2Y6 receptor‐dependent microglial phagocytosis of synapses mediates synaptic and memory loss in aging
Source: Aging Cell. 2022 Dec 24;22(2):e13761. doi: 10.1111/acel.13761 (PMC9924939; doi:10.1111/acel.13761)

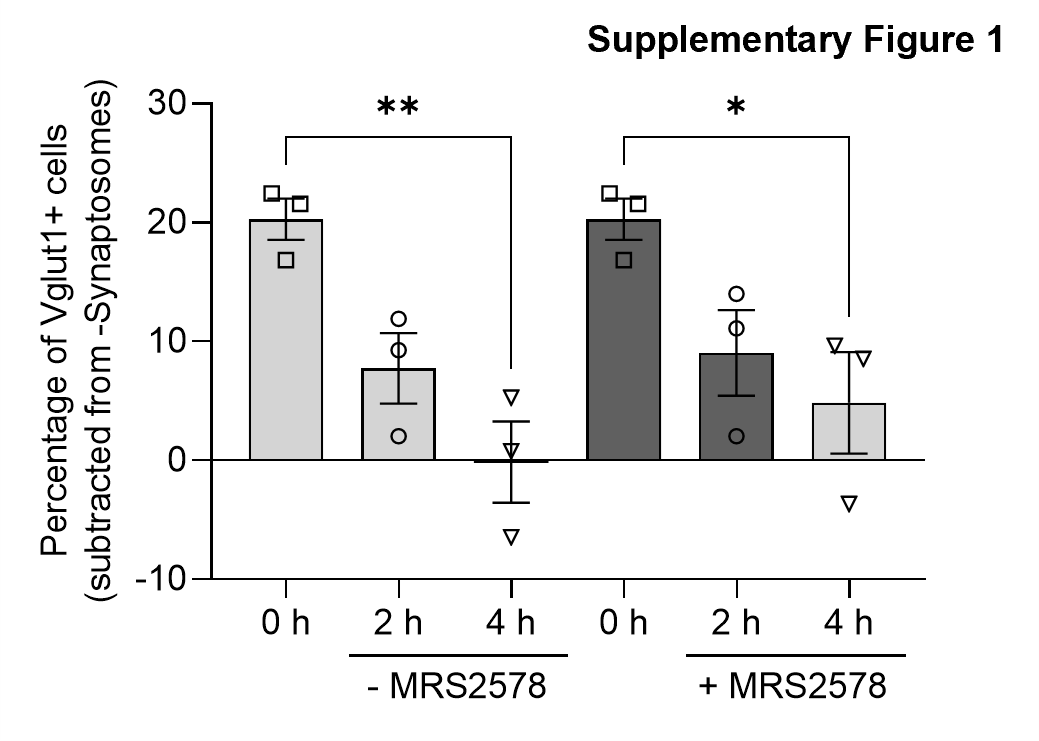

Supplement: Supplementary file 1 — Figure S1 [file ACEL-22-e13761-s002.tif]
