# Supplementary material for: P2Y6 receptor‐dependent microglial phagocytosis of synapses mediates synaptic and memory loss in aging
Source: Aging Cell. 2022 Dec 24;22(2):e13761. doi: 10.1111/acel.13761 (PMC9924939; doi:10.1111/acel.13761)
Supplement: Supplementary file 2 — Appendix S1 [file ACEL-22-e13761-s001.docx]

**Supplementary Figure 1: Microglial degradation of synaptosomes is P2Y_6_R independent.** Vglut1 staining was measured by flow cytometry 0, 2, and 4 hours (h) post-removal of synaptosomes from BV2 microglia (n=3), with and without 1 μM MRS2578, a P2Y_6_R inhibitor. 0 hour data is the same for ± MRS2578, but has been included twice to aid comparison. Each point represents one individual experiment with three technical replicates. Statistical comparisons were made via a two-way ANOVA with Bonferroni’s post-hoc comparisons. Error bars represent ± SEM, **P<0.01, *P<0.05.
